# Supplementary material for: Detailed molecular and epigenetic characterization of the pig IPEC-J2 and chicken SL-29 cell lines
Source: iScience. 2023 Feb 20;26(3):106252. doi: 10.1016/j.isci.2023.106252 (PMC10018572; doi:10.1016/j.isci.2023.106252)
Supplement: Data S1. Complete homer output for identified motifs in Pig IPECJ-2, related to Table 2 — Homer motif analysis results for histone modifications H3K4me1, H3K4me3, H3K27ac, and enhancer elements of pig IPECJ2 cell line. P-values >1e-10 are possible false positives. Within each folder (e.g. peak_files_CTCF) are the html files showing the identified motifs when using homer (e.g. homerResults.html). [file mmc2.zip › S5/Pig_IPECJ_2/peak_files_H3K4me3/homerResults/motif4.similar.html]

motif4

## Information for motif4

C
G
A
T
T
C
G
A
C
T
G
A
A
G
C
T
A
T
G
C
T
A
C
G
G
T
A
C
C
A
T
G
  
Reverse Opposite:  

G
T
A
C
C
A
T
G
A
T
G
C
T
A
C
G
T
C
G
A
G
A
C
T
A
G
C
T
G
C
T
A
  

|  |  |
| --- | --- |
| p-value: | 1e-158 |
| log p-value: | -3.649e+02 |
| Information Content per bp: | 1.587 |
| Number of Target Sequences with motif | 11616.0 |
| Percentage of Target Sequences with motif | 52.62% |
| Number of Background Sequences with motif | 11539.4 |
| Percentage of Background Sequences with motif | 43.61% |
| Average Position of motif in Targets | 150.5 +/- 86.1bp |
| Average Position of motif in Background | 149.5 +/- 104.1bp |
| Strand Bias (log2 ratio + to - strand density) | 0.0 |
| Multiplicity (# of sites on avg that occur together) | 1.46 |
| Motif File: | file (matrix) reverse opposite |

### Similar de novo motifs found

|  |  |  |  |  |  |  |  |
| --- | --- | --- | --- | --- | --- | --- | --- |
| Rank | Match Score | Redundant Motif | P-value | log P-value | % of Targets | % of Background | Motif file |
| 1 | 0.695 | T C A G T C G A T C G A A C G T C G T A T A C G G A T C C T A G T A C G T A G C | 1e-120 | -278.343101 | 69.98% | 62.46% | motif file (matrix) |
| 2 | 0.661 | A C G T A C G T C A G T G T C A T A G C A T C G T A G C A T C G T C G A A C G T | 1e-107 | -247.598694 | 37.42% | 30.45% | motif file (matrix) |
| 3 | 0.628 | G A T C T A G C C A T G C G T A A T C G G A C T A G C T A C G T C G T A G T A C | 1e-18 | -41.664158 | 0.78% | 0.37% | motif file (matrix) |
